# Clinical and MicroRNA Responses to Fecal Microbiota Transplantation in Patients with Alcohol-Related Cirrhosis: A Pilot Study

**DOI:** 10.3390/diagnostics16060846

**Published:** 2026-03-12

**Authors:** Cristian Ichim, Adrian Boicean, Samuel Bogdan Todor, Ioana Boeras, Paula Anderco, Victoria Birlutiu

**Affiliations:** Faculty of Medicine, Lucian Blaga University of Sibiu, 550169 Sibiu, Romania; cristian.ichim@ulbsibiu.ro (C.I.); ioana.boeras@ulbsibiu.ro (I.B.); paula.anderco@ulbsibiu.ro (P.A.); victoria.birlutiu@ulbsibiu.ro (V.B.)

**Keywords:** alcohol-related liver cirrhosis, fecal microbiota transplantation, microRNA, gut microbiota

## Abstract

**Background/Objectives**: Alcohol-related liver cirrhosis is a systemic disorder characterized by profound immune, metabolic and gut–liver axis dysregulation. Emerging evidence highlights a bidirectional interaction between the intestinal microbiota and host microRNAs (miRNAs), positioning this axis as a potential regulator of systemic homeostasis. However, human data exploring the impact of microbiota modulation on miRNA expression in advanced liver disease remain limited. **Methods**: Six patients with alcohol-induced liver cirrhosis underwent fecal microbiota transplantation (FMT). Safety was assessed through clinical and paraclinical monitoring at predefined intervals. Quality of life was evaluated pre- and post-intervention using a validated liver-specific questionnaire. Fecal expression of miR-21-5p, miR-122-5p, miR-125-5p, miR-146-5p and miR-155-5p was analyzed and correlations with clinical domains, demographic variables and hepatic encephalopathy severity were explored. **Results**: FMT was well tolerated, with no severe adverse events reported. Preliminary improvements were observed in total clinical score (3.22 [3.06–3.57] vs. 4.25 [4.20–4.26], *p* = 0.001) and in several quality-of-life domains, including abdominal symptoms, fatigue, systemic manifestations, activity and emotional function (*p* < 0.05), while worry/concern scores remained unchanged. miR-125 and miR-146 demonstrated consistent associations with clinical status both before and after FMT, whereas miR-21 correlated mainly with age and body mass index. Notably, miR-125 and miR-146 were also associated with post-FMT hepatic encephalopathy severity, supporting their potential value as molecular correlates of clinical response in this exploratory study. **Conclusions**: In this pilot study, FMT appeared safe and was temporally associated with improvements in clinical parameters in alcohol-related cirrhosis, alongside dynamic changes in fecal miRNA expression. These preliminary findings support a potential microbiota–miRNA interaction and warrant validation in larger, controlled longitudinal studies.

## 1. Introduction

Chronic liver disease remains a major global health burden, with liver cirrhosis representing the final common pathway of progressive hepatic injury and accounting for substantial morbidity, mortality and healthcare utilization worldwide [1,2]. Alcohol-related liver cirrhosis, in particular, continues to pose significant clinical challenges due to its complex pathophysiology, multisystem involvement and limited therapeutic options once decompensation occurs [3,4]. Beyond structural hepatic damage, cirrhosis is increasingly recognized as a systemic disorder, characterized by immune dysregulation, chronic inflammation, metabolic alterations, neurocognitive impairment, and profound disturbances in gut–liver axis homeostasis [5].

In recent years, mounting evidence has highlighted the pivotal role of the intestinal microbiota in the progression and complications of chronic liver disease [6]. Alterations in gut microbial composition, commonly referred to as dysbiosis, have been implicated in increased intestinal permeability, bacterial translocation, systemic inflammation, and the development of cirrhosis-related complications such as hepatic encephalopathy, ascites and infections [7]. The causes of microbiota alteration can be multiple and drug therapies are often also involved [8,9]. These findings have positioned the gut microbiota as both a key pathogenic driver and a promising therapeutic target in liver disease, leading to the exploration of specific microbiota-modulating therapies [10]. Consequently, fecal microbiota transplantation (FMT) has emerged as an innovative intervention aimed at restoring microbial balance and modulating host immune responses, with encouraging results reported in selected cirrhotic populations [11].

Parallel to advances in microbiome research, microRNAs (miRNAs) have gained increasing attention as critical post-transcriptional regulators of gene expression, involved in the control of inflammatory pathways, immune responses, metabolic regulation and cellular stress adaptation [12,13]. In the context of liver disease, specific miRNAs have been associated with hepatic inflammation, fibrosis progression, carcinogenesis and neurocognitive dysfunction [14,15]. Moreover, miRNAs are increasingly recognized as stable, measurable biomarkers with potential diagnostic, prognostic and therapeutic relevance [16]. However, despite their considerable theoretical promise, miRNA-based therapeutic strategies have yet to achieve robust clinical translation, largely due to challenges related to delivery, specificity and safety.

Emerging experimental and clinical data suggest a complex, bidirectional interaction between the intestinal microbiota and host miRNA expression [17]. Microbial metabolites and inflammatory signals can influence miRNA transcription, while host-derived miRNAs may, in turn, modulate microbial composition and intestinal immune responses [17,18,19]. This reciprocal relationship positions the microbiota–miRNA axis as a key integrative regulator of systemic homeostasis [20]. Nevertheless, human data regarding the impact of targeted microbiota modulation, such as FMT, on microRNA expression patterns in advanced liver disease remain limited. To date, no human studies have systematically characterized fecal microRNA responses following fecal microbiota transfer in patients with liver cirrhosis.

Against this background, the present study was designed to explore the clinical, molecular and safety-related implications of FMT in patients with alcohol-induced liver cirrhosis. Specifically, we aimed to evaluate the safety and tolerability of FMT in this vulnerable population, assess changes in patient-reported clinical and quality-of-life scores following the intervention and investigate the associations between fecal miRNA expression profiles and clinical outcomes before and after microbiota transfer. By integrating clinical assessments with molecular analysis, this study seeks to provide novel insights into miRNA–microbiota interactions as potential molecular correlates and indicators of therapeutic response in chronic liver disease.

## 2. Materials and Methods

### 2.1. Study Population and Eligibility Criteria

This pilot study enrolled adult patients admitted to the Sibiu County Clinical Emergency Hospital, primarily within the Department of Gastroenterology, who had a confirmed diagnosis of alcohol-related (ethanol-induced) liver cirrhosis. Eligibility was not restricted by Child–Pugh classification. However, participants were required to demonstrate verified alcohol abstinence following fecal microbiota transplantation (FMT), as confirmed by a close relative or caregiver, which led to the exclusion of several candidates. Despite this selection criterion, no significant differences in baseline characteristics were observed across the different Child–Pugh severity groups. Exclusion criteria included age below 18 years, non-alcoholic etiologies of cirrhosis, uncertain or unverified diagnoses, and the presence of concurrent oncological or major traumatic conditions that could influence study outcomes. Patients who declined participation, failed to provide written informed consent, or presented with coma or severe cognitive impairment were also excluded. Participants enrolled during the study period were all male, reflecting the natural recruitment outcome rather than a predefined inclusion criterion. This homogeneous sex distribution may reduce biological variability related to sex-dependent differences in microbiota composition and immune–metabolic responses.

Six patients with liver cirrhosis selected for FMT underwent comprehensive baseline evaluations, which included clinical assessment and stool sample collection for microRNA analysis. A full colonoscopy to the cecum was performed and FMT was administered at this site using material obtained from a healthy donor. The six patients with ethanol-induced liver cirrhosis who underwent FMT were closely monitored to assess both safety and efficacy. Follow-up assessments were conducted at 6 h and approximately one week after transplantation, with minor adjustments based on patient availability and institutional logistics. These assessments included clinical examination, abdominal ultrasonography and collection of an additional stool sample. Throughout the study period, both alcohol consumption and the use of any antibiotics, including rifaximin, were strictly prohibited.

Hepatic encephalopathy was assessed using the EncephalApp v2.0.7—2023—Stroop Test, a tool widely recognized for its high sensitivity in detecting minimal hepatic encephalopathy, a condition that is often difficult to identify through routine clinical evaluation. Encephalopathy staging was further supported by correlating cognitive test performance with clinical examination findings, in accordance with the West Haven classification.

### 2.2. Fecal Donor Selection and Preparation

Fecal donors were recruited from patients’ relatives or unrelated volunteers. Selection criteria included young age, absence of comorbidities and provision of written informed consent. Donors underwent thorough clinical and laboratory screening to minimize the risk of pathogen transmission. Additionally, a standardized epidemiological questionnaire was used to identify and exclude candidates with potential infectious or transmissible risks relevant to patients with liver cirrhosis. On the day of transplantation, donors prepared the fecal suspension according to a standardized protocol, adapted from procedures routinely used in the Sibiu Gastroenterology Clinic for Clostridioides difficile infection, and strictly followed in all cases. Stool samples collected on the morning of the procedure were processed within six hours to preserve microbial viability. At least 70 g of fecal material was homogenized with 250 mL of 0.9% sodium chloride solution for three minutes, then filtered through two to three layers of gauze. The resulting filtrate was used for transplantation. Colonoscopy was performed following standard bowel preparation with polyethylene glycol-based solutions, and all procedures were conducted under analgosedation. Patients were instructed to avoid bowel evacuation for at least two hours after the procedure to enhance microbiota engraftment. No additional medications potentially affecting study outcomes were administered during the study period.

### 2.3. RNA Extraction and miRNA Quantification by RT-qPCR

Given the concurrent evaluation of fecal microRNA expression, the study explored potential correlations between pre-procedural clinical status and concentrations of specific microRNAs associated with chronic liver disease, as well as between post-procedural microRNA levels and patients’ clinical status approximately one week after transplantation. This analysis holds significant scientific value, as notable post-interventional variations were observed both in clinical outcomes and in microRNA expression profiles.

Total RNA was extracted from the samples using TRIzol Reagent (Invitrogen/ThermoFisher Scientific, Waltham, MA, USA) following the manufacturer’s instructions, and RNA was resuspended in a final volume of 100 µL of ultrapure water. The quantity and quality of the extracted miRNA were assessed by fluorometry using the Qubit miRNA Assay kit and a Qubit 4 Fluorometer (ThermoFisher Scientific, Waltham, MA, USA). For the quantification of selected miRNAs, the TaqMan Advanced miRNA Assay kit (ThermoFisher Scientific, Waltham, MA, USA) was employed. This assay consists of two main steps: cDNA synthesis and miRNA quantification by real-time PCR. The cDNA synthesis procedure involves multiple steps, including polyadenylation, adaptor ligation, reverse transcription, and PCR amplification of the RT products. All steps were performed according to the manufacturer’s protocol, starting with 2 µL of total RNA per sample. Quantification of the target miRNAs was then carried out by real-time PCR using specific primers and a FAM-TAMRA-labeled probe, the TaqMan Fast Advanced Master Mix, and 5 µL of cDNA template on a QuantStudio 5 thermocycler (Applied Biosystems by ThermoFisher Scientific, Waltham, MA, USA). Cycling conditions consisted of an initial denaturation at 95 °C for 20 s, followed by 40 cycles of 95 °C for 1 s and 60 °C for 20 s. All amplifications were performed in triplicate for each sample. The laboratory workflow was meticulously followed, ensuring that all procedures adhered strictly to the manufacturer’s protocols. The TaqMan™ Advanced methodology was selected to provide a simpler and more reliable comparative evaluation than the classical method, which carries a slightly higher risk of error. The entire process was designed to allow accurate quantitative assessment of miRNA levels obtained from stool samples using RT-qPCR.

The microRNAs analyzed included were:

- hsa-miR-21-5p
- hsa-miR-122-5p
- hsa-miR-125-5p
- hsa-miR-146-5p
- hsa-miR-155-5p.

### 2.4. Assessment of Patients’ Quality of Life

Objective assessment of patients’ quality of life was conducted using a well-established and internationally validated scoring system with over 25 years of clinical application. This instrument was originally developed by Younossi and colleagues and published in Gut in 1999 [21]. Based on a questionnaire of considerable conceptual complexity, the tool has undergone multiple reinterpretations and methodological adaptations over time by various authors. However, in the present study, the original version, recognized for the robustness of its clinical validation, was employed. The only modification consisted of adjusting the reference period for responses from two weeks to one week, in accordance with the experimental design.

The original version is distinguished by its scientific rigor, clarity in item formulation and optimal patient accessibility, thereby providing a reliable and reproducible measure of quality of life. Accordingly, patients responded to a total of 29 questions, with the initial assessment also administered prior to microbiota transplantation. The questionnaire included the following items:

1. How often during the past week were you bothered by abdominal bloating?
2. How often did you feel tired or exhausted during the past week?
3. How often did you experience body pain during the past week?
4. How often did you feel sleepy during the day in the past week?
5. How often did you experience abdominal pain during the past week?
6. How often did shortness of breath interfere with your daily activities during the past week?
7. How often were you unable to eat as much as you would have liked during the past week?
8. How often were you bothered by reduced physical strength during the past week?
9. How often did you have difficulty lifting or carrying heavy objects during the past week?
10. How often did you feel anxious during the past week?
11. How often did you have low energy levels during the past week?
12. How often did you feel unhappy during the past week?
13. How often did you feel drowsy during the past week?
14. How often were you bothered by dietary restrictions during the past week?
15. How often did you feel irritable during the past week?
16. How often did you have difficulty sleeping at night during the past week?
17. How often were you bothered by abdominal discomfort during the past week?
18. How often did you worry about the impact of liver disease on your family during the past week?
19. How often did you experience mood changes during the past week?
20. How often were you unable to fall asleep at night during the past week?
21. How often did you experience muscle cramps during the past week?
22. How often did you worry that your symptoms would worsen during the past week?
23. How often did you experience dry mouth during the past week?
24. How often did you feel depressed during the past week?
25. How often did you worry that your condition would deteriorate during the past week?
26. How often did you have difficulty concentrating during the past week?
27. How often were you bothered by itching during the past week?
28. How often did you worry that you would never feel better during the past week?
29. How often did you worry about the availability of a liver in case of liver transplantation during the past week?

Responses to all questions were selected from seven possible options:

1. All of the time
2. Most of the time
3. A good part of the time
4. Some of the time
5. A little of the time
6. Hardly ever
7. None of the time
8. Domains and Items

The 29 items were grouped into domains representing specific dimensions of quality of life, allowing for more in-depth evaluation and localization of clinical issues. These domains were defined as follows:

- Abdominal Symptoms (AS): Items 1, 5, 17
- Fatigue (FA): Items 2, 4, 8, 11, 13
- Systemic Symptoms (SS): Items 3, 6, 21, 23, 27
- Activity (AC): Items 7, 9, 14
- Emotional Function (EF): Items 10, 12, 15, 16, 19, 20, 24, 26
- Worry/Concern (WO): Items 18, 22, 25, 28, 29

The authors recommend presenting questionnaire results on a standardized scale ranging from 1 to 7 by dividing the total score for each domain by the corresponding number of items. This approach enables uniform and comparable interpretation of results across different studies and populations.

### 2.5. Statistical Analysis

Given the small sample size (*n* = 6), continuous variables were characterized using measures of central tendency and dispersion appropriate for non-parametric distributions. Accordingly, data are expressed as median and interquartile range (IQR), defined as the interval between the 25th and 75th percentiles. Pre- and post-FMT clinical scores were compared using the Wilcoxon signed-rank test, selected for its suitability in paired, non-normally distributed data with small sample sizes. This non-parametric test evaluates whether the distribution of differences between paired observations is symmetric around zero, without assuming normality of the underlying data.

The associations between fecal microRNA expression levels and clinical domain scores, demographic variables (age, BMI), Child–Pugh class and hepatic encephalopathy severity were assessed using Pearson’s correlation coefficient (r). Correlation results are presented as r values alongside corresponding two-tailed *p*-values.

Statistical significance was defined as *p* < 0.05 for all analyses. Significance thresholds were classified as follows: * *p* < 0.05, ** *p* < 0.01, and *** *p* < 0.001.

## 3. Results

### 3.1. Adverse Events and Safety Profile

**Figure 1:**
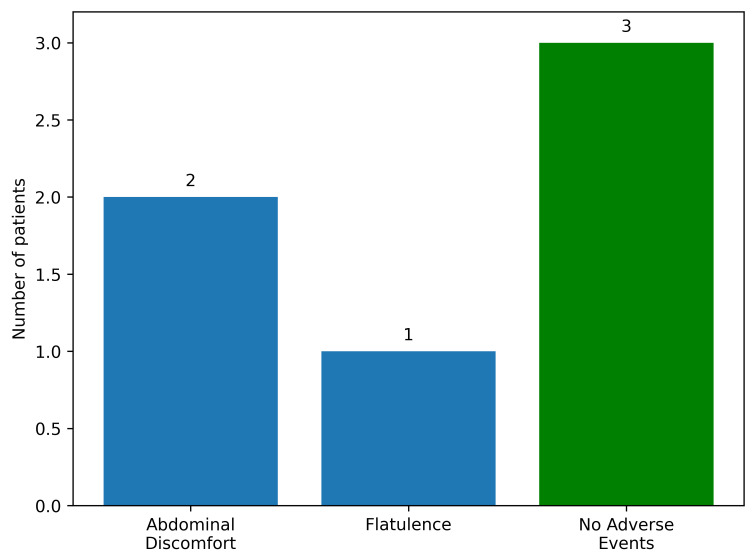
Distribution of immediate adverse events following fecal microbiota transplantation.

From a safety perspective, paraclinical assessments did not reveal major alterations that could pose a life-threatening risk to patients following FMT. No medium- or long-term adverse events were identified, and no colonoscopy-related complications occurred (including perforation, hemorrhage or systemic infections). However, mild immediate adverse reactions were observed within the first 6 h post-intervention. The most relevant immediate reactions are illustrated in Figure 1.

### 3.2. Analysis of Clinical Scores Before and After Fecal Microbiota Transplantation

The comparison of clinical scores before and after FMT was performed using the Wilcoxon signed-rank test, considering the paired nature of the data and their non-parametric distribution. A statistically significant improvement in the total clinical score was observed after FMT (*p* = 0.001), indicating an overall beneficial effect of the intervention.

Additionally, statistically significant increases were recorded for the abdominal, fatigue, systemic, activity, and emotional domains following FMT (all *p* < 0.05), highlighting a favorable impact of the intervention on clinical symptomatology and patient-reported quality of life (Table 1).

In contrast, the worry domain did not show significant changes between the two assessment time points (*p* = 0.527), reflecting stability in this dimension. Overall, the data support the effectiveness of FMT in improving most of the analyzed clinical parameters.

### 3.3. Pre-FMT Clinical Scores and MicroRNA Expression Analysis

Subsequently, correlations between microRNA expression levels and domain-specific clinical scores were assessed according to the methodological framework described in Section 2. Pearson correlation analysis revealed multiple significant associations between pre-FMT microRNA expression and clinical scores.

Elevated levels of miR-125 and miR-146 were negatively correlated with the abdominal symptom score (r = −0.710, *p* = 0.010 and r = −0.711, *p* = 0.010, respectively), indicating that higher expression of these microRNAs was associated with reduced severity of abdominal manifestations.

Furthermore, miR-21 demonstrated a very strong negative correlation with the worry score (r = −0.961, *p* < 0.001), suggesting that increased expression of this microRNA was associated with lower perceived anxiety levels.

**Figure 2:**
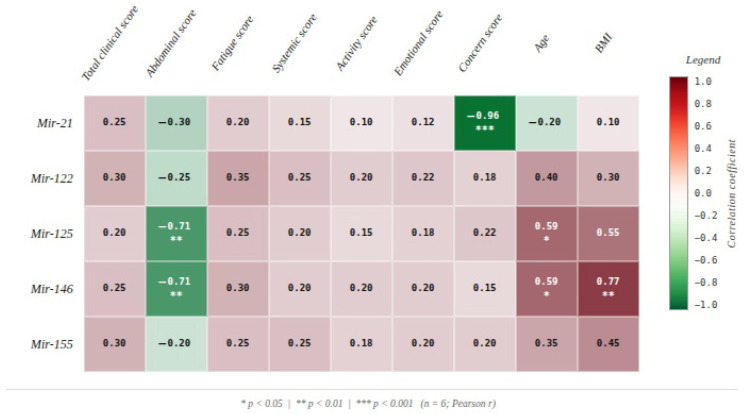
Pearson heatmap of correlation between pre-FMT microRNA expression levels (Mir-21, Mir-122, Mir-125, Mir-146, Mir-155) and clinical outcome measures including domain-specific symptom scores, age, and BMI (*n* = 6). Color intensity reflects the magnitude of the correlation coefficient (red = positive, green = negative). Asterisks denote statistical significance: * *p* < 0.05, ** *p* < 0.01, *** *p* < 0.001.

In addition, age was positively correlated with the expression levels of miR-125 and miR-146 (r = 0.594, *p* = 0.042 and r = 0.595, *p* = 0.041, respectively), while body mass index (BMI) showed a significant positive correlation with miR-146 expression (r = 0.765, *p* = 0.004) (Figure 2).

### 3.4. Post-FMT Clinical Scores and MicroRNA Expression Analysis

Analysis of the relationship between post-FMT microRNA expression levels, clinical scores and demographic variables (age and BMI) revealed several associations of scientific relevance. Post-FMT expression of miR-125 was negatively and significantly correlated with the total clinical score (r = −0.585, *p* = 0.046), suggesting that higher levels of this microRNA were associated with lower overall symptom burden and with observed improvements in clinical parameters following the intervention.

A similar pattern was observed for fatigue, where post-FMT miR-125 showed a strong negative correlation with the fatigue score (r = −0.786, *p* = 0.002), indicating a potential association between increased miR-125 expression and reduced post-interventional fatigability.

Conversely, post-FMT miR-146 exhibited a significant positive correlation with the abdominal symptom score (r = 0.734, *p* = 0.007), suggesting a possible role in maintaining visceral reactivity. At the emotional level, post-FMT miR-146 was significantly negatively correlated with the emotional score (r = −0.637, *p* = 0.026), potentially reflecting a modulatory effect on post-interventional affective response.

**Figure 3:**
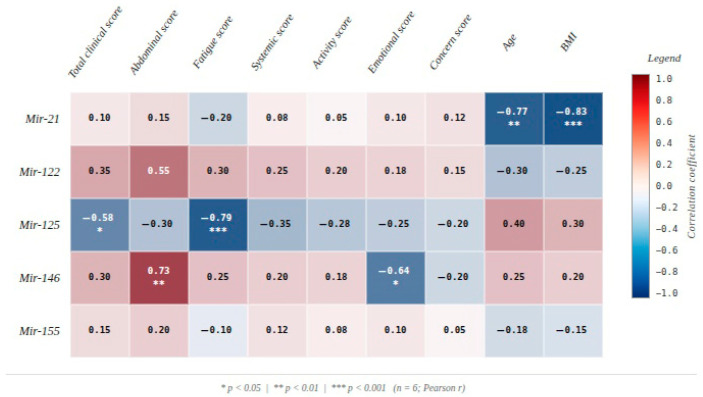
Pearson heatmap of correlation between post-FMT microRNA expression levels (Mir-21, Mir-122, Mir-125, Mir-146, Mir-155) and clinical outcome measures including domain-specific symptom scores, age, and BMI (*n* = 6). Red indicates positive correlations, blue indicates negative correlations. Asterisks denote statistical significance: * *p* < 0.05, ** *p* < 0.01, *** *p* < 0.001.

Regarding demographic variables, post-FMT miR-21 showed significant negative correlations with both age (r = −0.771, *p* = 0.003) and BMI (r = −0.829, *p* = 0.001). These strong associations suggest that miR-21 expression tends to decrease progressively with advancing age and increasing BMI, possibly reflecting cumulative effects of these factors on epigenetic and molecular regulatory mechanisms (Figure 3).

Overall, the results indicate that post-FMT miR-125 and miR-146 exhibited the strongest associations with post-interventional clinical scores, suggesting that they may represent molecular correlates of the observed biological changes following FMT. In contrast, post-FMT miR-21 appears to be predominantly associated with clinico-demographic and anthropometric variables, particularly age and BMI, potentially reflecting systemic influences on microRNA expression.

### 3.5. Child–Pugh Class, Post-FMT Encephalopathy, and MicroRNA Expression

In the analysis of correlations between post-FMT microRNA expression and clinical parameters, Child–Pugh class did not show significant associations with any of the analyzed microRNAs, with correlation coefficients remaining low and statistically non-significant.

In contrast, the severity of post-FMT hepatic encephalopathy was significantly associated with specific microRNAs. miR-125 demonstrated a moderate and statistically significant positive correlation (r = 0.648, *p* = 0.023), suggesting that higher expression levels may be linked to increased encephalopathy severity. Conversely, miR-146 showed a significant negative correlation with encephalopathy severity (r = −0.649, *p* = 0.022), indicating an inverse association with neurological complication severity, without implying causality.

**Figure 4:**
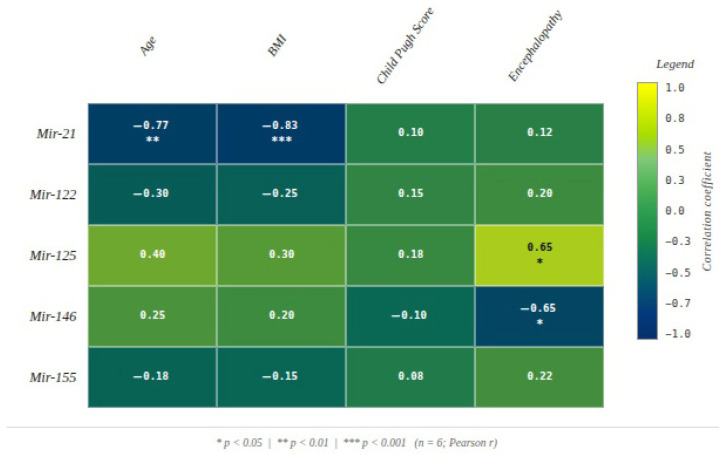
Pearson heatmap of correlation between post-FMT microRNA expression levels (Mir-21, Mir-122, Mir-125, Mir-146, Mir-155) and hepatic clinical parameters including Child–Pugh score, encephalopathy severity, age, and BMI (*n* = 6). Yellow indicates strong positive correlations, dark blue indicates strong negative correlations. Asterisks denote statistical significance: * *p* < 0.05, ** *p* < 0.01, *** *p* < 0.001.

No significant associations were observed between the remaining microRNAs and the analyzed parameters (Figure 4).

## 4. Discussions

The relationship between the intestinal microbiota and miRNA expression has long attracted scientific interest, based on the premise that both components are closely linked to a broad spectrum of digestive and extra-digestive conditions, whose trajectories, favorable or unfavorable, may influence each other bidirectionally. Conclusive evidence has emerged primarily in recent years, through rigorous studies objectively demonstrating a bidirectional interplay between the microbiota and miRNAs, thereby highlighting their complementary roles in the regulation of homeostasis and in the pathogenesis of chronic diseases [22,23,24]. Nevertheless, to substantiate this concept further, researchers systematically explored whether alterations in microbiota composition can, in turn, induce changes in miRNA expression. The results were compelling: the interaction is indeed bidirectional, with miRNAs not only being shaped by microbiota shifts, but also actively contributing to microbial regulation, including via modulation of intestinal inflammatory processes [25].

This framework not only clarifies the implication of miRNAs in disorders such as ulcerative colitis or colorectal neoplasia, but also opens innovative research avenues regarding whether microbiota compositional changes may directly or indirectly shape miRNA expression. The significance of this hypothesis is fundamental, as even indirect influences on microbial equilibrium could generate complex systemic effects with meaningful implications for maintaining organismal homeostasis and finely modulating pathophysiological responses.

Its relevance becomes even greater considering that miRNA-targeted therapies, despite their strong theoretical promise and sustained efforts over recent decades, have not yet progressed beyond the experimental stage. The lack of robust clinical validation and the absence of approved therapeutics underscore the complexity of molecular-level interventions and support the need to reconsider indirect pathways, such as microbiota modulation, as potentially safer therapeutic tools. In this context, targeting intestinal microflora may represent a viable and safer alternative for achieving effects analogous to those intended by miRNA-based therapies [26].

From a procedural safety standpoint, the present findings confirm that FMT is an intervention with an excellent tolerability profile when performed under strictly controlled conditions and with careful donor selection. The stability of paraclinical parameters and the absence of severe complications are consistent with the existing literature, which reports an extremely low rate of major adverse events associated with this approach [27]. The lack of severe events in this study, such as perforation, hemorrhage or systemic infection, further supports the safety of endoscopic delivery via colonoscopy, provided that pre-procedural preparation protocols are rigorously followed and post-interventional surveillance is carefully implemented. Moreover, this favorable safety profile may be attributed both to comprehensive microbiological donor screening and to continuous clinical monitoring of recipients, which represent essential safeguards for procedural success and optimal tolerability. According to recent literature, the risk of systemic infection or bacteremia related to FMT is extremely low and has been reported almost exclusively in patients with compromised immune status or severe comorbid pathology [28,29].

The mild reactions observed in the first hours after the procedure, such as abdominal discomfort or bloating, fall within the transient symptom profile reported in other comparable clinical studies. These manifestations most likely reflect the physiological process of intestinal adaptation to a newly introduced microbial ecosystem and are self-limiting, typically resolving spontaneously within approximately 24 h [30]. Collectively, these results strengthen the current evidence base, confirming that FMT has a favorable safety profile comparable to other therapeutic endoscopic procedures and can be applied safely in a closely monitored clinical setting. Furthermore, the absence of late adverse events during follow-up suggests effective integration of the transplanted microbiota, without clinically meaningful systemic disruption of patient homeostasis.

In the pre-transfer stage, significant negative correlations were identified between miR-125 and miR-146 expression levels and the abdominal symptom score, suggesting that higher expression of these miRNAs is associated with reduced digestive symptom severity. These findings are consistent with published data describing miR-125b and miR-146 as negative regulators of intestinal inflammatory signaling, capable of inhibiting NF-κB pathway activation and reducing secretion of pro-inflammatory cytokines, particularly IL-6 and TNF-α [31,32]. In addition, miR-21 showed a strong negative correlation with the worry score, indicating that higher expression of this molecule is associated with lower levels of anxiety and perceived stress. This observation is supported by literature highlighting the role of miR-21 in neuroendocrine adaptation to stress, through modulation of the hypothalamic–pituitary–adrenal axis and regulation of cellular responses to psychophysiological stimuli [33,34,35]. Age and BMI also correlated with miR-125 (positive correlation) and miR-146, in line with evidence implicating these miRNAs in chronic inflammatory processes and immune regulation associated with metabolic alterations [36,37].

The analysis of post-transfer miRNA levels in relation to clinical scores indicates a dynamic evolution of the molecular profile. Post-FMT miR-125 showed a significant negative correlation with both the total clinical score (r = −0.585, *p* = 0.046) and, more strongly, the fatigue score (r = −0.786, *p* = 0.002). These findings suggest that higher post-interventional miR-125 levels are associated with global clinical improvement and reduced fatigability, pointing toward a potential involvement in systemic recovery processes and homeostatic regulation following microbiota transfer. In contrast, post-FMT miR-146 demonstrated a significant positive correlation with the abdominal score (r = 0.734, *p* = 0.007), which may indicate a transient reactivation of compensatory inflammatory mechanisms during the integration and adaptation phase of the new microbiota. Simultaneously, miR-146 exhibited a significant negative correlation with the emotional score (r = −0.637, *p* = 0.026), which may suggest a protective contribution to post-interventional emotional regulation, consistent with controlled neuroinflammation models described by Zhao and colleagues [38].

With respect to demographic variables, post-FMT miR-21 correlated negatively with both age (r = −0.771, *p* = 0.003) and BMI (r = −0.829, *p* = 0.001), suggesting a progressive reduction in miR-21 expression with advancing age and increasing body mass. This trend may reflect reduced regenerative activity and diminished adaptive cellular responsiveness in the context of aging-related metabolic and inflammatory shifts. Overall, the results indicate that miR-125 and miR-146 represent the principal molecular correlates of post-FMT clinical scores, supporting their potential value as candidate biomarkers associated with favorable clinical response following fecal microbiota transfer. By contrast, miR-21 appears to be more tightly linked to patients’ demographic and metabolic profiles, potentially reflecting systemic regulatory particularities. These observations support the concept of a complex recalibration of an immune–microbial–emotional axis in the post-interventional period, through which normalization of miRNA expression may contribute to restoration of homeostatic balance both intestinally and at the psycho-neuro-vegetative level.

The absence of significant correlations between Child–Pugh class and miRNA expression levels suggests that these biomolecules may not directly reflect the global degree of structural hepatic dysfunction, at least within the post-FMT monitoring interval analyzed. The literature does report correlations between certain miRNAs and Child–Pugh class, but predominantly in the context of hepatocellular carcinoma development in chronic hepatitis B infection, rather than in cirrhosis per se [39]. In contrast, post-FMT hepatic encephalopathy severity exhibited significant correlations with specific miRNAs, opening an important perspective regarding the role of these RNA molecules in neuro-hepatic regulation. Notably, miR-125 showed a moderate, statistically significant positive correlation (r = 0.648, *p* = 0.023) with encephalopathy severity, suggesting that elevated miR-125 levels may be associated with more pronounced neurocognitive dysfunction. Although the precise pathophysiological mechanism remains incompletely clarified, available evidence supports plausible hypotheses linking miR-125 to interactions among systemic inflammation, blood–brain barrier integrity, and ammonia metabolism. Specifically, miR-125 has been shown to participate in astrocytic functional regulation and neuronal communication, processes disrupted by toxin accumulation in cirrhosis, as well as in modulation of oxidative stress responses. This dual involvement may explain the observed association between increased miR-125 expression and hepatic encephalopathy severity [40,41,42]. The finding that miR-125 levels increase with high-grade encephalopathy may also reflect a compensatory response to oxidative stress-driven dysfunction, suggesting that this molecule could exert a protective role within redox imbalance and neuronal adaptation. Thus, elevated miR-125 expression may represent an endogenous defense reaction aimed at mitigating neurotoxic effects of accumulated metabolites in advanced hepatic dysfunction.

Conversely, miR-146 showed a significant negative correlation (r = −0.649, *p* = 0.022) with encephalopathy grade, suggesting a potential protective role in maintaining functional neuronal communication. miR-146 is well documented in multiple neurological disorders, particularly those involving neuroinflammatory mechanisms, where it exerts both regulatory and protective functions. In this setting, reduced miR-146 expression may reflect a secondary effect of ammonia toxicity on neural tissue, via impairment of molecular defense and neuronal regulatory pathways. The absence of other significant correlations between the remaining miRNAs and analyzed parameters may be attributable to the relatively small sample size and to etiological heterogeneity of hepatic injury in the studied cohort. Nevertheless, the present findings provide a solid starting point for the hypothesis that miR-125 and miR-146 may serve as valuable complementary biomarkers for assessing the risk and severity of hepatic encephalopathy, particularly in the context of microbiota-targeted interventions such as FMT.

## 5. Conclusions

This exploratory, single-arm study suggests that FMT can be safely administered in patients with alcohol-induced liver cirrhosis under strict clinical supervision and rigorous donor selection. The absence of severe adverse events and the transient nature of mild post-procedural symptoms support the procedural feasibility of this approach in a carefully monitored setting.

Improvements observed in overall clinical status and multiple quality-of-life domains following FMT should be interpreted with caution in the absence of a control group, as natural disease fluctuation, regression to the mean, or hospitalization-related stabilization cannot be excluded. Nevertheless, the consistent directionality of changes across several clinical domains provides a signal warranting further controlled investigation.

Molecular analysis identified dynamic associations between fecal microRNA expression and post-interventional clinical trajectories. miR-125 and miR-146 emerged as potential correlates of clinical evolution, suggesting a possible biomarker role, while miR-21 appeared more strongly associated with demographic and metabolic characteristics.

Although limited by small sample size and the absence of a comparator arm, this pilot study generates mechanistic and clinical hypotheses supporting the rationale for adequately powered randomized controlled trials designed to clarify the efficacy of FMT and to validate fecal microRNAs as candidate biomarkers in microbiota-based interventions for chronic liver disease.

## Figures and Tables

**Table 1:** Pre- and post-FMT variation in clinical scores.

Parameter	Pre-FMT Median (IQR)	Post-FMT Median (IQR)	*p*-Value
Total clinical score	3.22 (3.06–3.57)	4.25 (4.20–4.26)	0.001 *
Abdominal score	3.33 (3.00–4.00)	4.67 (4.33–5.00)	0.002 *
Fatigue score	3.00 (2.80–3.20)	3.80 (3.60–4.00)	0.001 *
Systemic score	3.70 (3.20–4.60)	4.70 (4.60–5.20)	0.021 *
Activity score	3.50 (3.00–3.66)	5.17 (5.00–5.33)	0.001 *
Emotional score	3.25 (3.13–3.50)	3.88 (3.75–4.13)	0.005 *
Worry/Concern score	2.80 (2.60–3.20)	3.00 (2.80–3.20)	0.527

* *p* < 0.05.

## Data Availability

The data presented in this study are available from the corresponding author upon reasonable request due to privacy, legal and ethical reasons.
